# Supplementary material for: Evolutionary Rates, Divergence Rates, and Performance of Individual Mitochondrial Genes Based on Phylogenetic Analysis of Copepoda
Source: Genes (Basel). 2023 Jul 22;14(7):1496. doi: 10.3390/genes14071496 (PMC10379994; doi:10.3390/genes14071496)
Supplement: Supplementary file 1 [file genes-14-01496-s001.zip › fig S3.pdf]

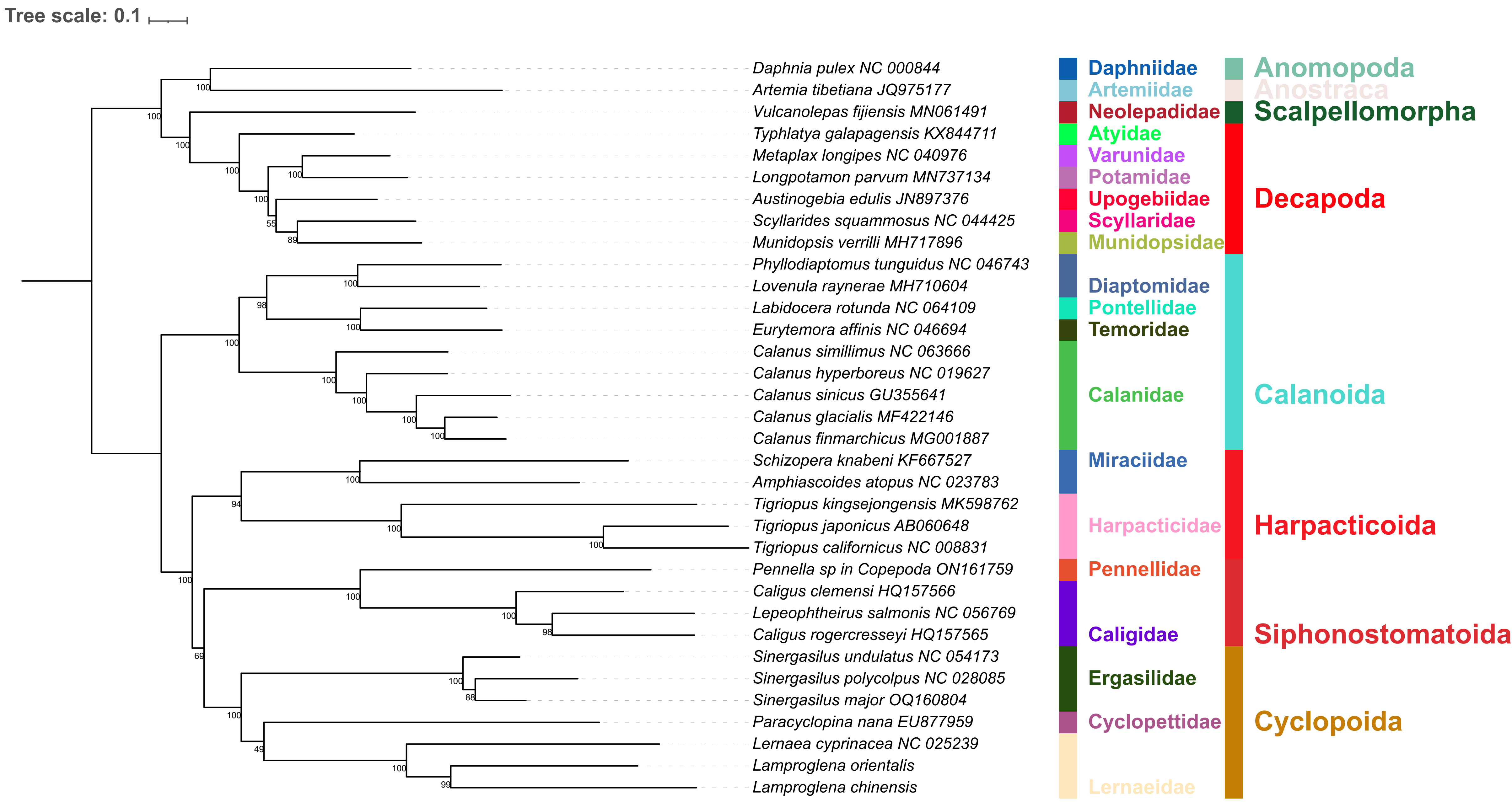

subgroup_1 partitioned ML

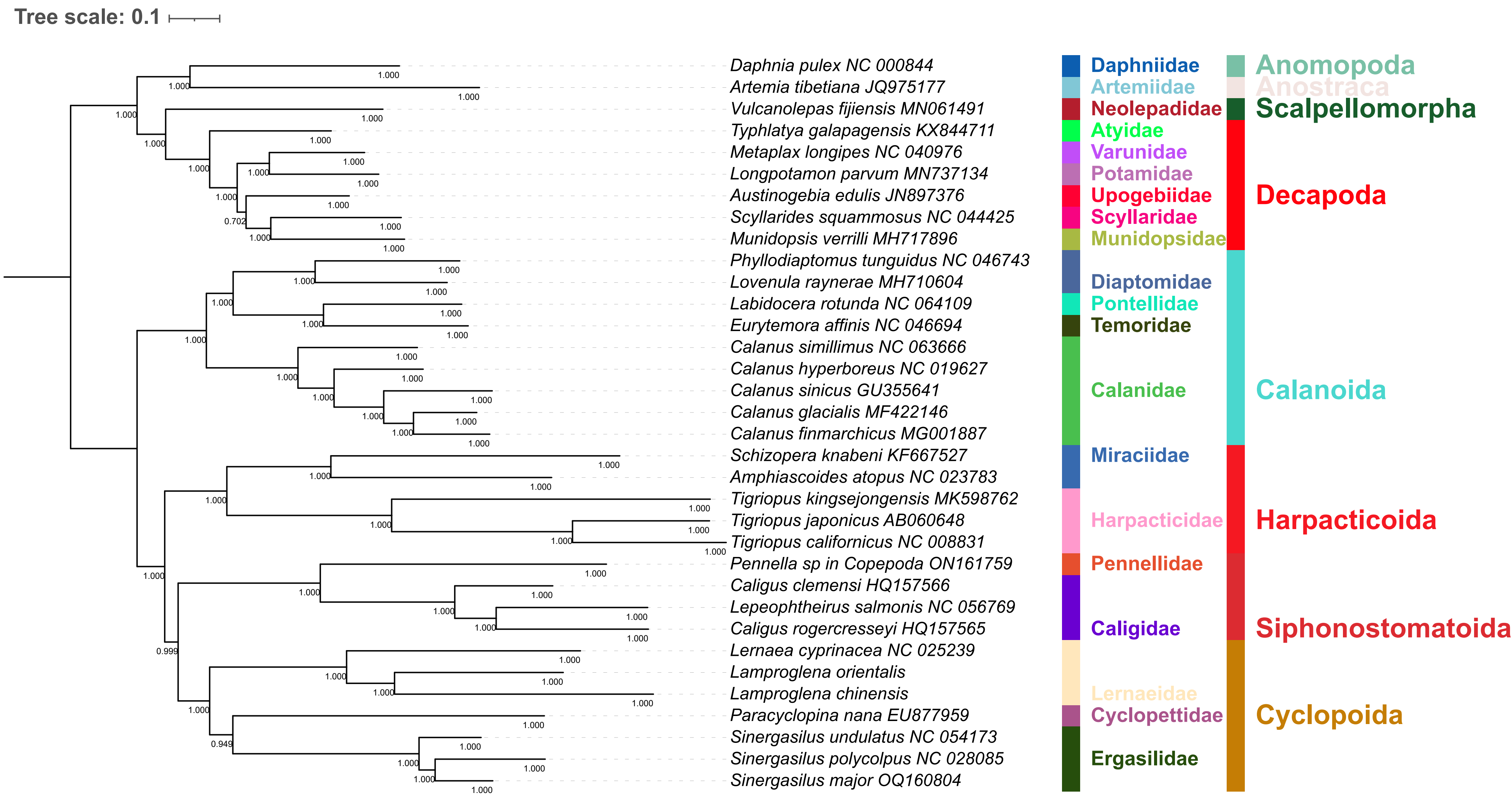

subgroup_1 partitioned BI

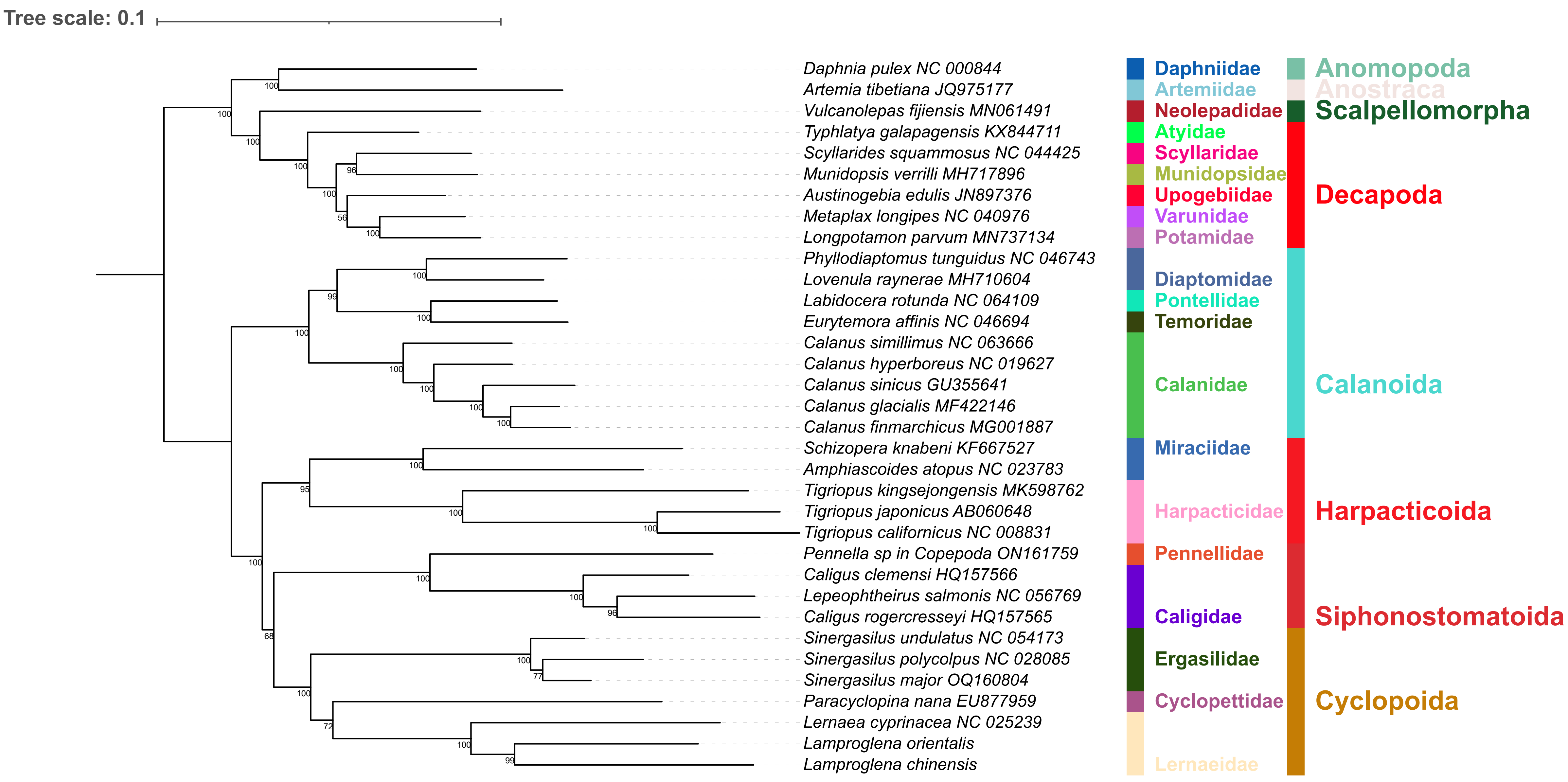

subgroup_1 unpartitioned ML

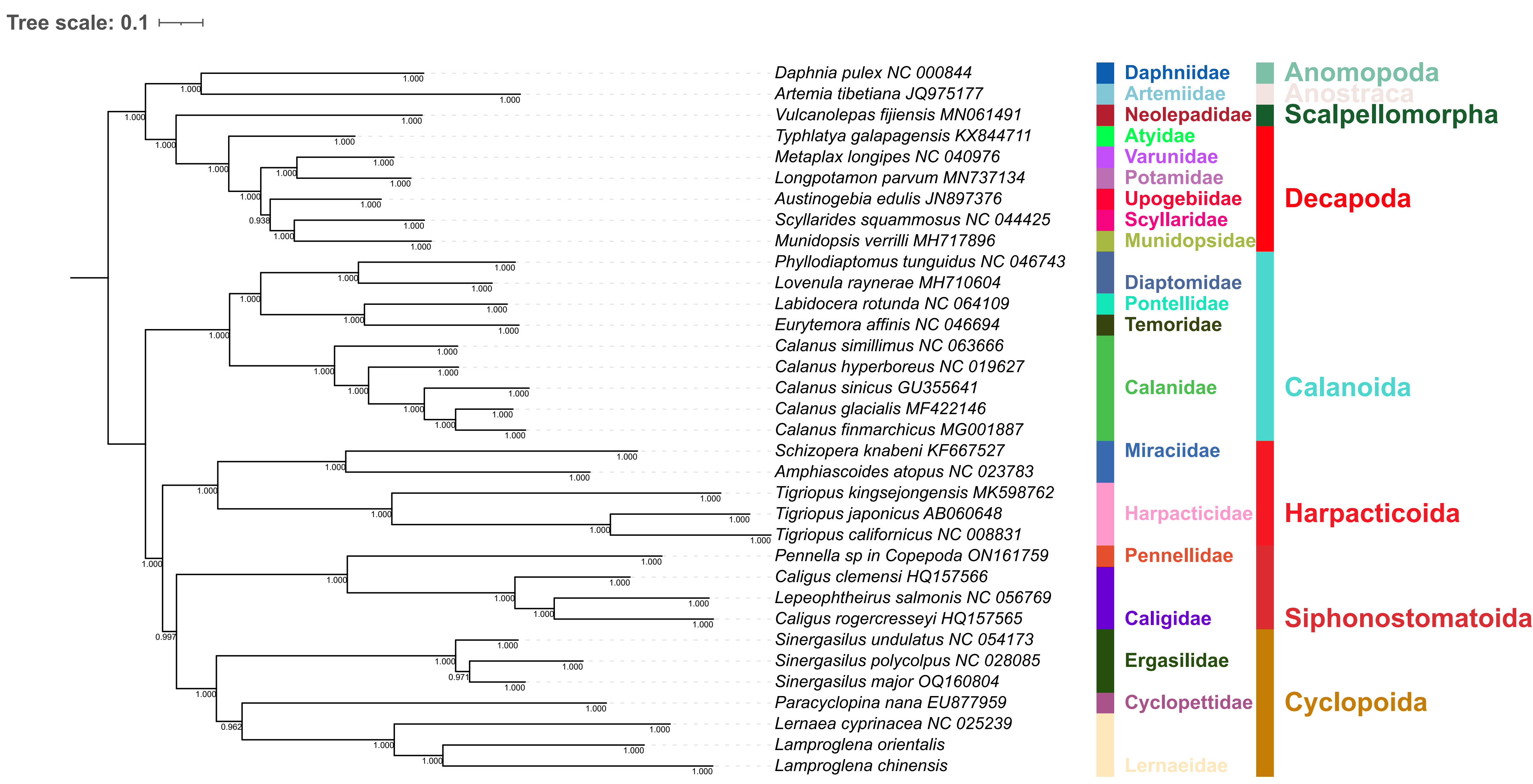

subgroup_1 unpartitioned BI

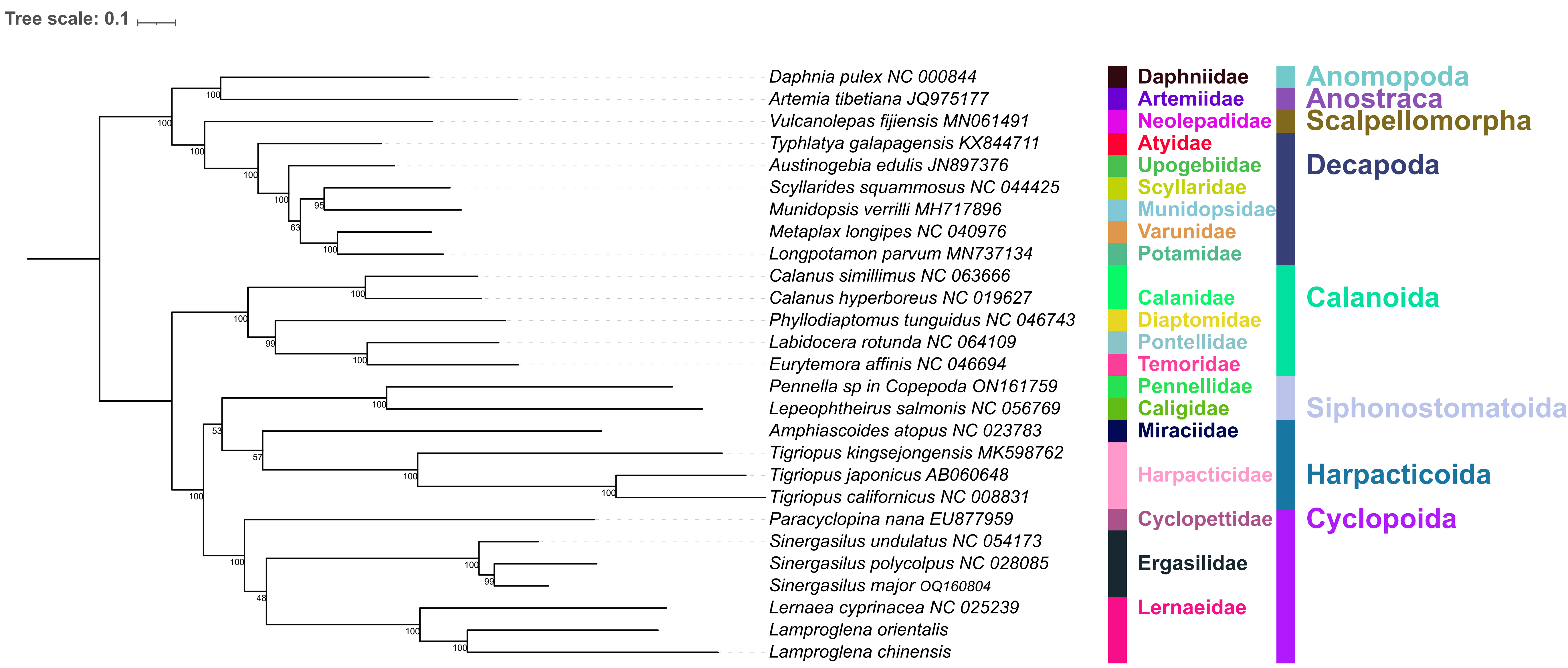

subgroup_2 partitioned ML

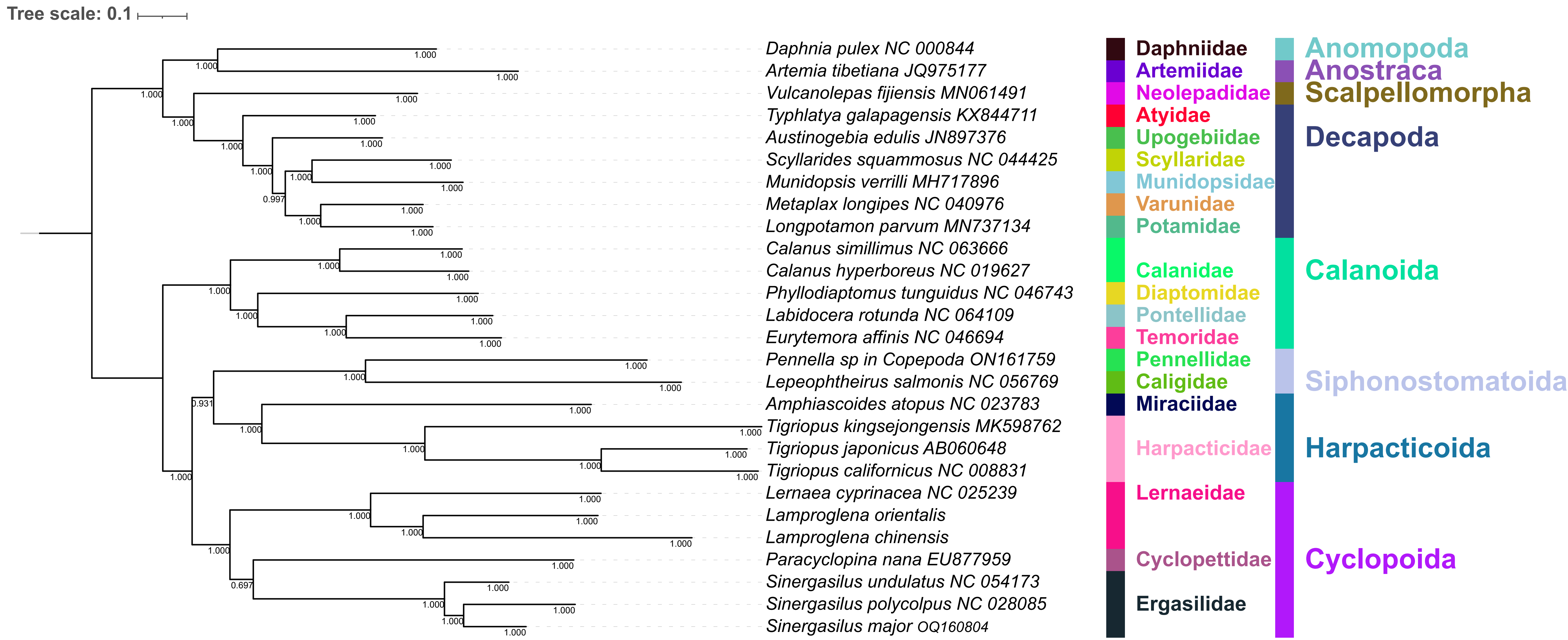

subgroup_2 partitioned BI

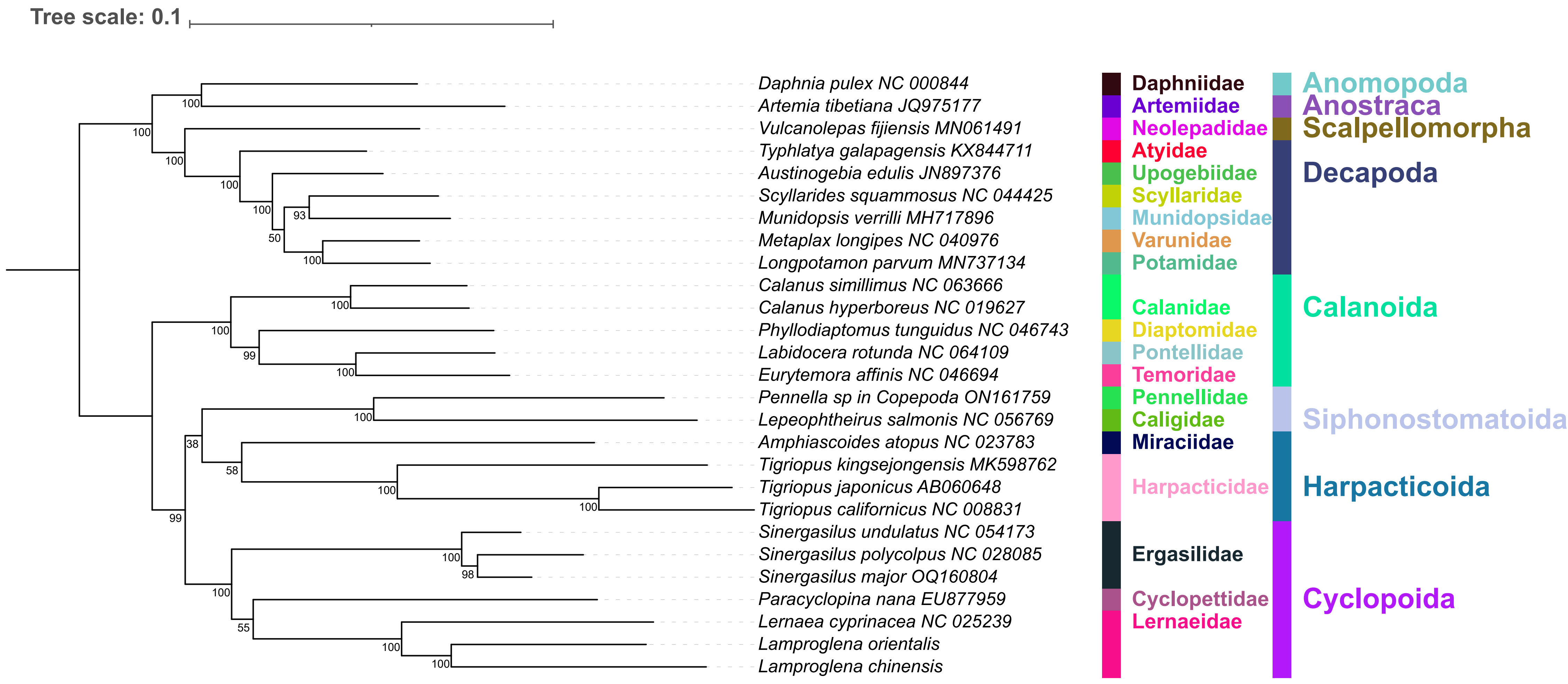

subgroup_2 unpartitioned ML

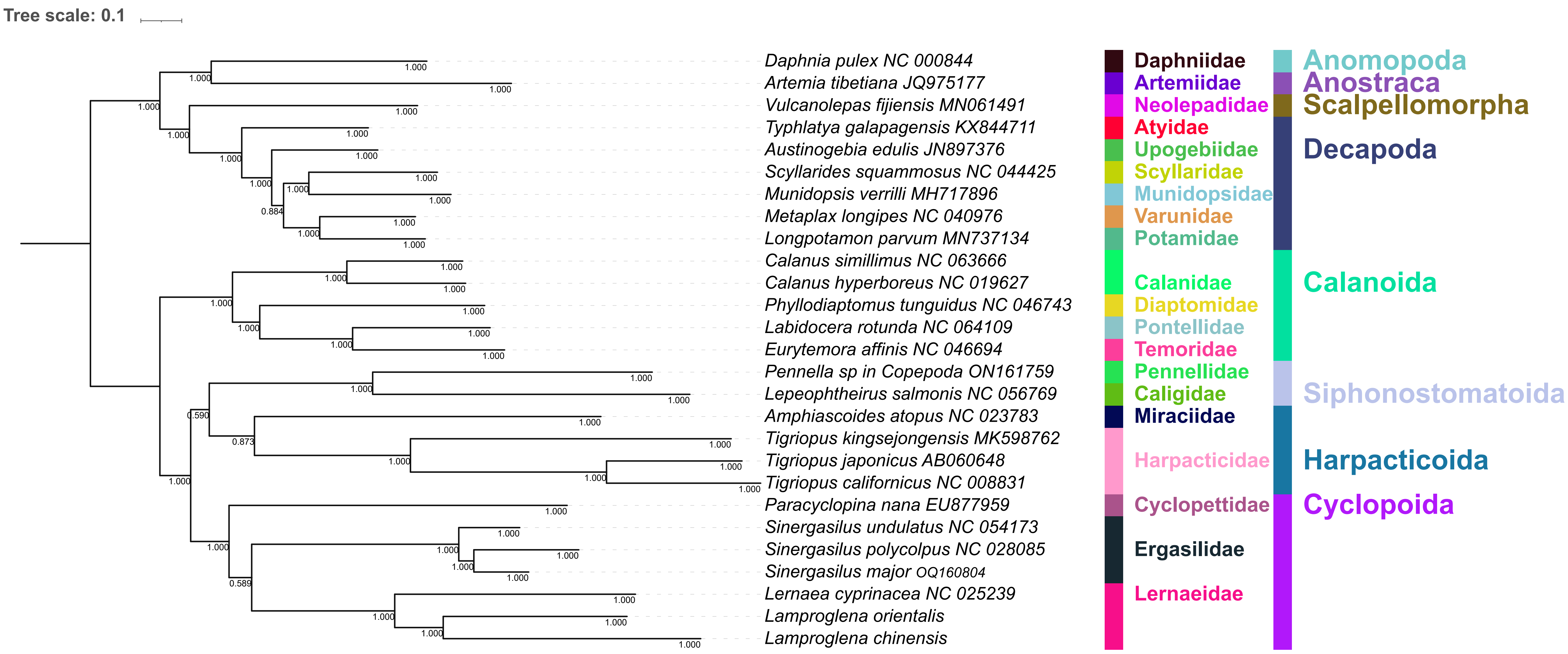

subgroup_2 unpartitioned BI
