# Supplementary figures and images for: Evolutionary Rates, Divergence Rates, and Performance of Individual Mitochondrial Genes Based on Phylogenetic Analysis of Copepoda
Source: Genes (Basel). 2023 Jul 22;14(7):1496. doi: 10.3390/genes14071496 (PMC10379994; doi:10.3390/genes14071496)

# *Sinergasilus major*

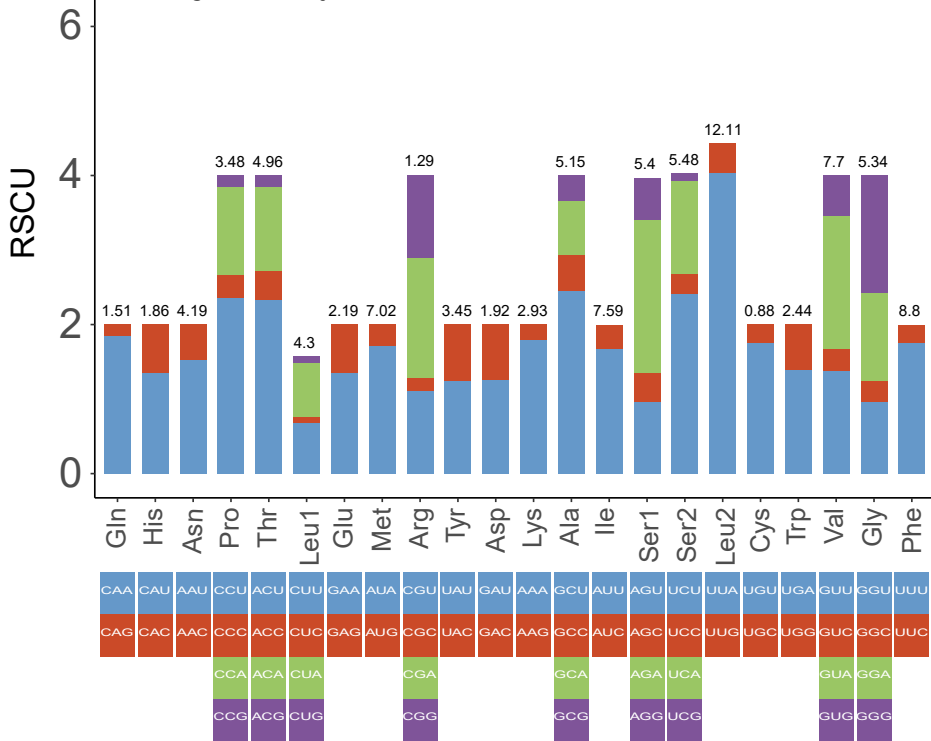

Supplement: Supplementary file 1 [file genes-14-01496-s001.zip › fig. S1.pdf]

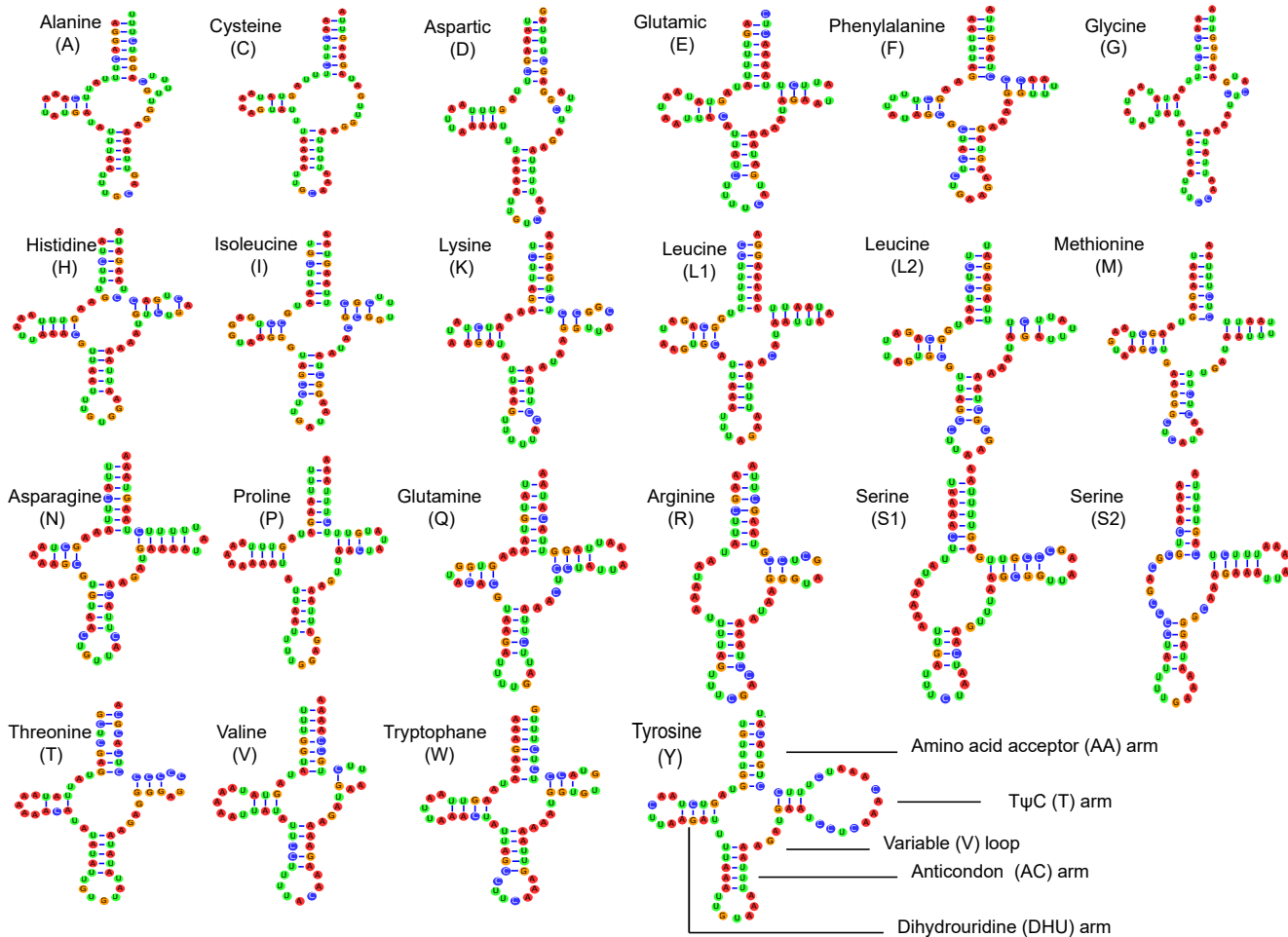

Supplement: Supplementary file 1 [file genes-14-01496-s001.zip › fig. S2.pdf]
